# Evaluation of United States chiropractic professional subgroups: a survey of randomly sampled chiropractors

**DOI:** 10.1186/s12913-021-07081-0

**Published:** 2021-10-05

**Authors:** Jordan A. Gliedt, Stephen M. Perle, Aaron A. Puhl, Sarah Daehler, Michael J. Schneider, Joel Stevans

**Affiliations:** 1 Department of Neurosurgery, Medical College of Wisconsin, Milwaukee, WI USA; 2 University of Bridgeport, College of Health Sciences, School of Chiropractic, Bridgeport, CT USA; 3 Murdoch University, Discipline of Chiropractic, College of Science, Health, Engineering and Education, Murdoch, Western Australia Australia; 4 Private Practice, Able Body Health Clinic, Lethbridge, AB Canada; 5 Department of Physical Therapy, University of Pittsburgh, Pittsburgh, PA USA; 6 University of Pittsburgh, Clinical Translation and Science Institute, Pittsburgh, PA USA

**Keywords:** Chiropractic, Healthcare integration, Healthcare teams, Attitude of health personnel, Interprofessional relations, Professional identity

## Abstract

**Background:**

Professional subgroups are common and may play a role in aiding professional maturity or impeding professional legitimization. The chiropractic profession in the United States has a long history of diverse intra-professional subgroups with varying ideologies and practice styles. To our knowledge, large-scale quantification of chiropractic professional subgroups in the United States has not been conducted. The purpose of this study was to quantify and describe the clinical practice beliefs and behaviors associated with United States chiropractic subgroups.

**Methods:**

A 10% random sample of United States licensed chiropractors (*n* = 8975) was selected from all 50 state regulatory board lists and invited to participate in a survey. The survey consisted of a 7-item questionnaire; 6 items were associated with chiropractic ideological and practice characteristics and 1 item was related to the self-identified role of chiropractic in the healthcare system which was utilized as the dependent variable to identify chiropractic subgroups. Multinomial logistic regression with predictive margins was used to analyze which responses to the 6 ideology and practice characteristic items were predictive of chiropractic subgroups.

**Results:**

A total of 3538 responses were collected (39.4% response rate). Respondents self-identified into three distinct subgroups based on the perceived role of the chiropractic profession in the greater healthcare system: 56.8% were spine/neuromusculoskeletal focused; 22.0% were primary care focused; and 21.2% were vertebral subluxation focused. Patterns of responses to the 6 ideologies and practice characteristic items were substantially different across the three professional subgroups.

**Conclusions:**

Respondents self-identified into one of three distinct intra-professional subgroups. These subgroups can be differentiated along themes related to clinical practice beliefs and behaviors.

**Supplementary Information:**

The online version contains supplementary material available at 10.1186/s12913-021-07081-0.

## Introduction

Though professional membership provides many universally binding characteristics representative of its overall membership, the existence of professional subgroups with differing ideologies and characteristics is common [1, 2]. Differences between healthcare professional subgroups may range from subtle differences to vast divergences in ideologies and practice characteristics. These differences among subgroups may act as either a vehicle for healthy professional evolution or, at times, as an impediment toward professional growth [2].

Chiropractic is a profession with a long history of divisive intra-professional subgroups with wide variations in ideologies and practice styles [2–4]. A major ongoing source of conflict within the chiropractic profession in the United States has centered on the scope or role of the chiropractor within the healthcare system [5]. It is likely that there are at least two unique chiropractic subgroups within the United States, defined by their perceived roles: 1) a spine and musculoskeletal focused subgroup [6–9] and 2) a subgroup concentrating on vertebral subluxation detection and removal [7, 10]. However, a previous report of the chiropractic profession conducted by the Institute for Alternatives Futures (IAF) suggests that there are three chiropractic subgroups within the United States: 1) a group focused on correcting spinal subluxations to free the body’s self-healing capacity; 2) a group focused on spine and musculoskeletal conditions; and 3) a group focused on primary care or specialties dealing with a range of conditions beyond the spine [11].

Chiropractic is presently the largest complementary and alternative health profession in the United States, and acceptance into mainstream health care systems is steadily increasing [3]. The United States is the origin of the chiropractic profession and contains the largest number of practicing chiropractors in the world [12]. To our knowledge, the professional subgroupings and characteristics of chiropractic subgroups within the United States have not been quantified on a large-scale.

The purpose of this study was to expand upon prior studies which have quantified and described chiropractic subgroups internationally [2, 5, 13] and to explore chiropractic intra-professional associations [14–18] within the United States. This study specifically aimed to quantify and further describe characteristics associated with United States chiropractic subgroups – as similarly suggested in the IAF report [11]. We hypothesized a relationship between ideology, beliefs, and practice patterns of United States chiropractors indicating the best role for chiropractic was subluxation detection and removal, spine/neuromusculoskeletal care, or general primary care. This study is designed to aid in characterizing the chiropractic profession in the United States and inform stakeholders as to the unique features associated with chiropractic subgroup membership.

## Study design

This was a multi-stage survey of a randomly selected, stratified sample (by state and District of Columbia) of licensed chiropractors in the United States completed between March 2018 and January 2020.

## METHODS

### Survey Development

**Fig. 1:**
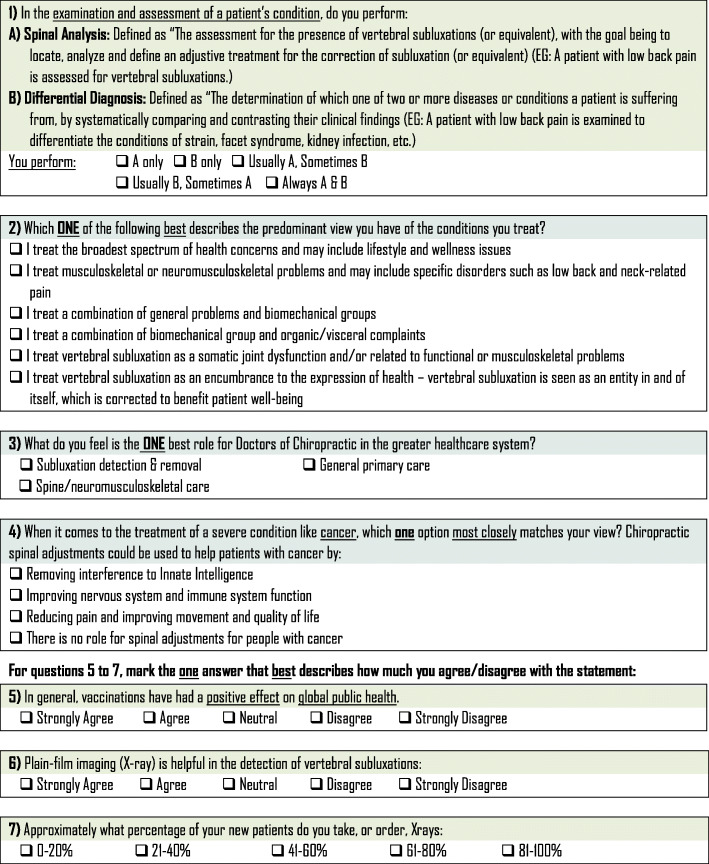
Ideology and practice behavior survey items

The survey instrument used in this study was developed by the authors and modeled after similar chiropractic identity analyses completed in Canada and Europe [2, 13]. The survey instrument included a total of 7 items intended to elicit clearly divergent ideologies and practice behaviors. Demographic information was also collected with the survey instrument. Items on the survey instrument solicited practice ideology and practice behavior information regarding clinical examination/assessment, health conditions treated, role of chiropractors in the healthcare system, the impact of chiropractic adjustments [spinal manipulation] in treating cancer patients, vaccinations attitudes, and x-ray use. Collection of demographic information included gender, state or district of primary practice, chiropractic program attended, and year of chiropractic program graduation. The survey instrument was piloted in a group of licensed chiropractors (*n* = 10) in Canada. The survey instrument was revised to improve clarity based on feedback provided during pilot testing, prior to administration to the randomly selected sample. Figure 1 is a copy of the survey instrument.

### Population sampling

The target population was actively licensed chiropractors in the United States. Those with a suspended or inactive chiropractic license to practice in one of fifty states in the United States or the District of Columbia, and those without a residence in the United States were excluded.

All fifty states and the District of Columbia licensing boards were contacted and a list of active licenses for each was obtained. Chiropractors with an active license from each state were numbered by their consecutive location on the list of licensees from each jurisdiction. A random number generator was used to select 10% of the total number from each list. The individual jurisdiction samples were combined. Duplicates, and chiropractors with an invalid mailing address, were removed yielding a total sample size of 8975 licensed Doctors of Chiropractic (DCs).

### Survey administration

This study used three stages of survey administration, resulting in a total of seven rounds of mailed invitations:

Stage 1 - For the first stage, an online survey and website was constructed and hosted by the University of Bridgeport. An invitation with instructions to complete the online survey was distributed by mail to each randomly selected participant in March 2018. Printed on each invitation was a unique random alphanumeric identifier code to obtain access to the survey online to ensure only one response. Responders were tracked and non-responders were mailed a follow up reminder to complete the online survey in April 2018 with a new abbreviated unique random alphanumeric identifier code and QR code to facilitate reaching the online survey. In June 2018, the online method to complete the survey was closed.

Stage 2 - The second stage of survey administration involved a mailed hard copy of the survey to be returned by mail. Each individual mailing included a letter inviting participation in the survey, a hard copy of the survey instrument, and a postage paid return addressed envelope. Envelopes included a variation of the phrase “Important Chiropractic Study” on the outside to entice opening of the invitation letter. Responders were tracked by use of a unique random alphanumeric identifier code embedded on the hard copy survey instrument in each mailing. Non-responders were mailed up to four follow up invitations between June 2018 and May 2019.

Stage 3 - The third stage involved an invitation to complete an abbreviated hard copy survey instrument in a postcard format to be returned by pre-paid postage mail. The invitation included instructions to complete the abbreviated survey instrument, and then tear off the completed survey instrument which had pre-paid postage to be returned by mail. This third stage was sent in January 2020 to non-responders. The abbreviated hard copy survey instrument included one item aimed to elicit practice ideology and behavior related to conditions treated (Fig. 1, Item #2) and two demographic items regarding the state of primary practice and chiropractic program attended. The practice ideology and behavior item included on the abbreviated survey instrument was chosen due to its prior survey use and importance in classifying chiropractors’ ideology and behavior [2, 13, 19]. Survey responses were terminated at the end of March 2020.

### Ethics

This study was approved by the University of Bridgeport Institutional Review Board (IRB ID: 2017-10-01). Invitations to complete the survey included a notice that all responses were anonymous. The invitation requested that anyone not wishing to complete the survey and to avoid being contacted again should return the survey in the postage paid envelope with a line through it. Those doing so were removed from further mailings. Survey completion was recorded in the database of the randomly selected chiropractors, so that after completing the survey no one would be sent the survey again. This was a strategy to prevent sharing of the survey with peers, in an effort to bias the results by asking like-minded chiropractors to participate. To ensure the anonymity of the respondents, a research assistant separated the envelopes (which may have had return addresses) from the survey instrument and discarded the envelopes. Then, she recorded that a response was received without recording the response itself. Only the first research assistant had access to the database of names and addresses and their associated unique identifier codes. The now deidentified surveys were delivered to one principal investigator (SMP) who maintained their security.

### Data management

Responses completed through the online mechanism were automatically uploaded to a protected electronic database. The deidentified responses returned through mail were entered manually into the electronic database by an independent pair of research assistants using a single-entry method by one research assistant with review and confirmation performed by the second research assistant. Any discrepancies were resolved by consensus. The hard copies of postal surveys were securely destroyed.

### Dependent variable

The dependent variable was chiropractic subgrouping, which consisted of three possible responses to the question “What is the ONE best role of chiropractic within the greater healthcare system?” (Fig. 1, Item #3). The responses to this question were: 1) Subluxation detection and removal; 2) General Primary Care; and 3) Spine/neuromusculoskeletal care. The three possible responses correspond with a prior suggestion of the potential for three chiropractic subgroups in the United States: 1) a subluxation focused subgroup; 2) a spine/neuromusculoskeletal focused subgroup; and 3) a “healthy life doctor” or general primary care focused subgroup [11].

### Independent variables

Independent variables included practice ideologies and behaviors related to clinical examination/assessment, health conditions treated, role of chiropractors in the healthcare system, the impact of chiropractic adjustments [spinal manipulation] in treating cancer patients, vaccination attitudes, and x-ray use (Fig. 1, Items 1, 2, 4, 5, 6, 7).

### Covariates

Covariates included demographic information. Demographic information included mode of survey completion, gender (male, female), years in practice, state of primary practice location, and chiropractic program attended.

### Statistical analysis

Statistical analyses were completed on aggregate data that was obtained from the entirety of the survey administration, including phase 1, phase 2, and phase 3. Statistical analysis was completed using STATA version 14 (StataCorp, College Station, TX, USA). Descriptive statistics were calculated for the dependent variable to identify chiropractic subgroups. Descriptive statistics were also calculated for each of the independent variables and demographic covariates.

This study tested the hypothesis that there are unique subgroups of United States DCs that can be distinguished from one another. Separate multinomial logistic regression models were performed to describe the relation between responses to the dependent variable and independent variables, controlling for all demographic covariates. To aid in the interpretation of the models, predictive margins plots were performed to analyze which responses to the independent variables were predictive of chiropractic subgroups.

## Results

**Fig. 2:**
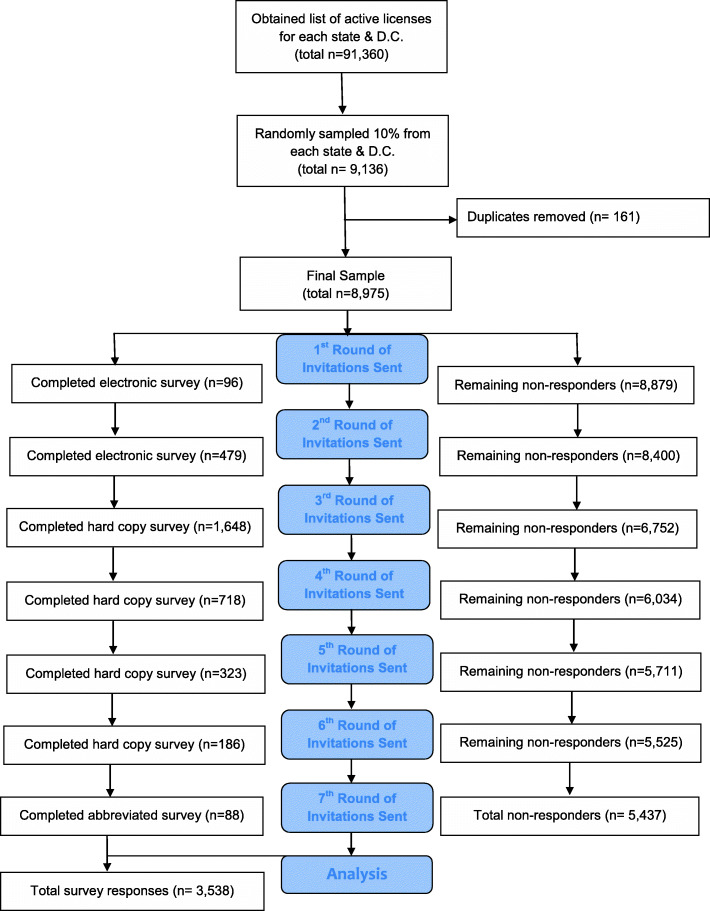
Study Protocol Flow Diagram

A total of 3538 responses across all 50 states and the District of Columbia were collected (39.4% response rate). The stages of survey administration and responses associated with each stage are summarized in Fig. 2.

### Demographic characteristics

The majority of respondents completed a full survey by mail delivery (80.7%). A small proportion of the sample completed the online (16.9%) or an abbreviated survey instrument via postcard (2.5%). Most respondents were male (74.6%) and almost evenly divided between 11 and 20, 21–30, and 31–40 years in practice (25.8, 25.7, and 23.8%, respectively). Demographic characteristics of respondents, including mode of survey completion, gender, and years in practice are shown in Table 1. Palmer College of Chiropractic – Iowa (20.8%) and Life University (12.0%) were the two most highly reported chiropractic programs attended among respondents. California was the most represented state (10.6%) among respondents. Total number of licensed chiropractors, survey mailings and response rates for each state and Washington, DC are shown in Additional file 1: Appendix 1A. A description of chiropractic programs attended by survey respondents are itemized in detail in Additional file 1: Appendix 1B.

**Table 1:** Sample demographics

Survey Type	Percentage
Online	16.9%
Mail	80.7%
Postcard	2.5%
**Gender Identification**	
Male	74.6%
Female	25.4%
Multiple	< 0.1%
**Years in Practice**	
1–10	17.3%
11–20	25.8%
21–30	25.7%
31–40	23.8%
> 40	7.4%

### Respondent subgroup analysis

**Fig. 3:**
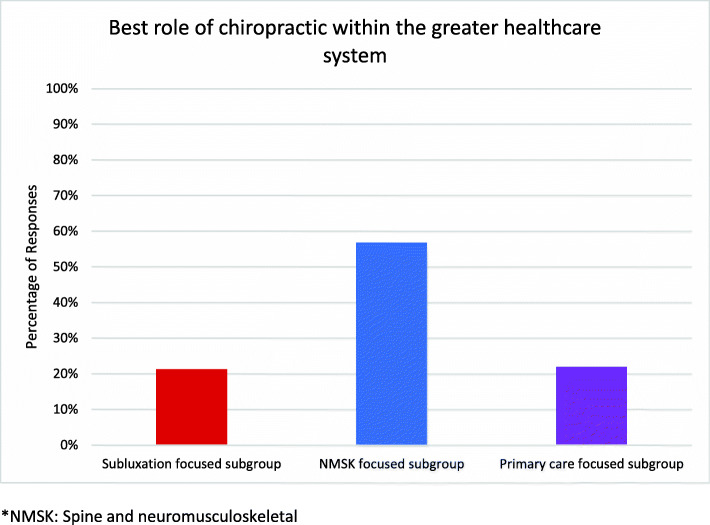
Percentages of responses to Survey Question 3: “What is the one best role of chiropractic within the greater healthcare system?” This question served as the dependent variable in all statistical analyses. *NMSK: Spine and neuromusculoskeletal

Overall sample percentages of responses to each of the survey items, the independent variables, are displayed in Additional file 1: Appendix 1C. Responses to the dependent variable, “What do you feel is the ONE best role for Doctors of Chiropractic in the greater healthcare system?” are illustrated in Fig. 3. The majority of respondents (56.8%) identified spine and neuromusculoskeletal care as the best role for the chiropractic profession within the greater healthcare system. The remainder chose general primary care (22.0%) or vertebral subluxation detection and removal (21.2%).

**Fig. 4:**
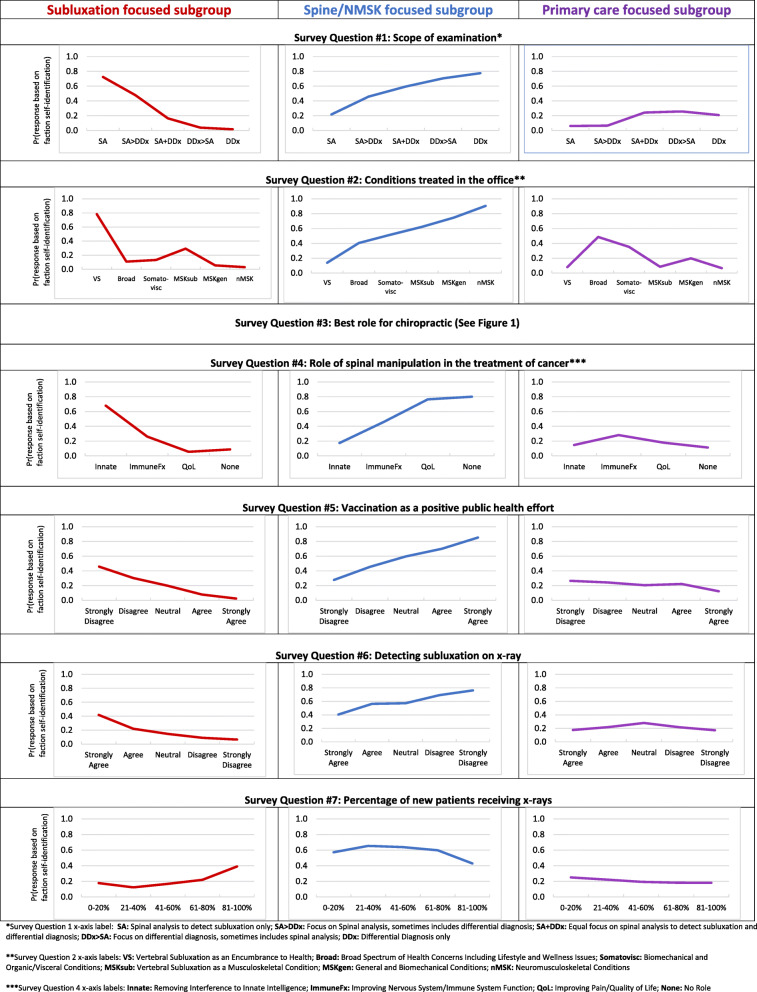
Survey question results as predictive margin plots illustrating the difference in attitudes toward the clinical assessment, examination, treatment, public health approach, and diagnostic imaging between three self-identified subgroups of US chiropractors; 1) Subluxation-focused; 2) Spine/neuromusculoskeletal-focused (NMSK); and 3) Primary care-focused. See Fig. 1 for survey questions in full. *Survey Question 1 x-axis label: **SA:** Spinal analysis to detect subluxation only; **SA > DDx:** Focus on Spinal analysis, sometimes includes differential diagnosis; **SA + DDx:** Equal focus on spinal analysis to detect subluxation and differential diagnosis; **DDx > SA:** Focus on differential diagnosis, sometimes includes spinal analysis; **DDx:** Differential Diagnosis only. **Survey Question 2 x-axis labels: **VS:** Vertebral Subluxation as an Encumbrance to Health; **Broad:** Broad Spectrum of Health Concerns Including Lifestyle and Wellness Issues; **Somatovisc:** Biomechanical and Organic/Visceral Conditions; **MSKsub:** Vertebral Subluxation as a Musculoskeletal Condition; **MSKgen:** General and Biomechanical Conditions; **nMSK:** Neuromusculoskeletal Conditions. ***Survey Question 4 x-axis labels: **Innate:** Removing Interference to Innate Intelligence; **ImmuneFx:** Improving Nervous System/Immune System Function; **QoL:** Improving Pain/Quality of Life; **None:** No Role

Multinomial logistic regression models revealed statistically significant differences between chiropractic subgroups and is shown in Additional file 1: Appendix 1D. Predictive margin plots demonstrating the association of the differing views and behaviors for each chiropractic subgroup are illustrated in Fig. 4. The predictive margin for a particular population characteristic represents the predicted probability of the outcome (which of the three subgroups the respondents self-selected) if every observation in the sample had that particular characteristic. For a specific example, refer to the predictive margin plot for survey question 1 in Fig. 4. If respondents reported the scope of their clinical examination to always include both a spinal analysis for the assessment of vertebral subluxation and a differential diagnosis, there was a 20% probability that those respondents belonged to the subluxation focused subgroup. Further interpretation of these respondents indicates they had a 60% probability of belonging to the spine and neuromusculoskeletal focused subgroup, and a 20% probability of belonging to the primary care focused subgroup.

Regarding scope of examination (survey question 1), respondents reporting the scope of their clinical examination to only include spinal analysis for the assessment of vertebral subluxation had a 70% probability of belonging to the subluxation focused subgroup, a 20% probability of belonging to the spine and neuromusculoskeletal focused subgroup, and a 10% probability of belonging to the primary care focused subgroup. Conversely, respondents who reported the scope of their clinical examination only includes a differential diagnosis had a 0% probability of belonging to the vertebral subluxation focused subgroup, an 80% probability of belonging to the spine and neuromusculoskeletal focused subgroup, and a 20% probability of belonging to the primary care focused subgroup.

Concerning conditions treated (survey question 2), respondents who reported predominantly treating vertebral subluxation as an encumbrance to health had an 80% probability of belonging to the vertebral subluxation focused subgroup, a 10% probability of belonging to the spine and neuromusculoskeletal focused subgroup, and a 10% probability of belonging to the primary care focused subgroup. In contrast, respondents reporting predominantly treating neuromusculoskeletal conditions had a 0% probability of belonging to the vertebral subluxation focused subgroup, a 90% probability of belonging to the spine and neuromusculoskeletal focused subgroup, and a 10% probability of belonging to the primary care focused subgroup.

Regarding the role of spinal manipulation for those with cancer (survey question 4), respondents reporting the role of spinal manipulation for those with cancer is to remove interference to innate intelligence had a 70% probability of belonging to the vertebral subluxation focused subgroup, a 20% probability of belonging to the spine and neuromusculoskeletal focused subgroup, and a 10% probability of belonging to the primary care focused subgroup. Respondents reporting there is no role of spinal manipulation in those with cancer also had a 10% probability of belonging to the subluxation focused subgroup, an 80% probability of belonging to the spine and neuromusculoskeletal focused subgroup, and a 10% probability of belonging to the primary care focused subgroup.

Regarding vaccination (survey question 5), respondents who strongly disagreed that vaccinations have had a positive effect on global public health had a 50% probability of belonging to the vertebral subluxation focused subgroup, an approximately 25% probability of belonging to the spine and neuromusculoskeletal focused subgroup, and an approximately 25% probability of belonging to the primary care focused subgroup. In contrast, respondents who strongly agreed that vaccinations have had a positive effect on global public health had a 0% probability of belonging to the vertebral subluxation focused subgroup, a 90% probability of belonging to the spine and neuromusculoskeletal focused subgroup, and a 10% probability of belonging to the primary care focused subgroup.

Concerning the detection of vertebral subluxation on x-ray (survey question 6), respondents who strongly agreed that x-ray is helpful in detecting vertebral subluxations had a 40% probability of belonging to the vertebral subluxation focused subgroup, a 40% probability of belonging to the spine and neuromusculoskeletal focused subgroup, and a 20% probability of belonging to the primary care focused subgroup. Respondents who strongly disagreed that x-ray is helpful in detecting vertebral subluxations had a near 0% probability of belonging to the vertebral subluxation focused subgroup, an 80% probability of belonging to the spine and neuromusculoskeletal focused subgroup, and slightly below a 20% probability of belonging to the primary care focused subgroup.

Regarding use of x-rays for new patients (survey question 7), respondents who reported prescribing x-rays for 0–20% of new patients had a 20% probability of belonging to the vertebral subluxation focused subgroup, a 60% probability of belonging to the spine and neuromusculoskeletal focused subgroup, and a 20% probability of belonging to the primary care focused subgroup. Respondents reporting prescribing x-rays for 81–100% of new patients had a 40% probability of belonging to the vertebral subluxation focused subgroup, a 40% probability of belonging to the spine and neuromusculoskeletal focused subgroup, and a 20% probability of belonging to the primary care focused subgroup.

## Discussion

This study provides data on a randomly sampled survey of licensed chiropractors (*n* = 3538) from all 50 states and the District of Columbia. This study tested associations between each subgroup and unique themes which have been identified as barriers to professional legitimization within mainstream healthcare systems, such as the scope of clinical examination, conditions treated, clinical practices for a non-musculoskeletal condition, vaccination views, and x-ray utilization. Results show clear differences in response patterns between each subgroup for each theme.

According to the IAF report, it is estimated that the majority of United States chiropractors (75–80%) fall within the spine and neuromusculoskeletal focused subgroup and can be characterized as providing care that is consistent with the role of a spine and musculoskeletal healthcare provider [11]. The results of this present study revealed a majority membership to the spine and neuromusculoskeletal subgroup, though less than IAF estimations, at nearly 57% of respondents. The IAF report also estimated a minority subgroup of chiropractors consistent with the primary care focused subgroup (10–15%) [11]. Findings of this present study show a slightly higher than estimated membership to this subgroup at 22% of respondents. Finally, the IAF report estimated about 10–15% of chiropractors fit into a vertebral subluxation focused subgroup. The results of this present study also show a slightly higher than estimated membership to this subgroup at 21% of respondents.

The majority of chiropractors in this study identified with the spine and neuromusculoskeletal subgroup, consistent with the IAF report. This identity is consistent with internal calls within the profession [20], longstanding public views and patient profiles [6, 21], and the ways the mainstream healthcare system has valued and integrated chiropractors [22–27]. Although findings suggest that the majority of United States chiropractors identify as spine and neuromusculoskeletal focused clinicians with higher probabilities of subscribing to a practice style more consistent with evidence, it appears that traditional beliefs that are inconsistent with modern scientific understanding, such as those related to vertebral subluxation, vaccination and x-ray utilization linger within the profession. These traditional beliefs may be barriers to professional legitimization within mainstream healthcare systems and are in need of further discussion, which will be addressed in more detail in a separate secondary paper.

It is possible that chiropractors could belong to multiple subgroups simultaneously. There is potential that chiropractors could subscribe to one or more traditional beliefs while also practicing in a manner which is more consistent with evidence in other areas. This is suggested by the data in this present study in which there were mixed subgroup views and practice patterns.

### Limitations and strengths

This study had multiple limitations. Neither cognitive interviewing nor psychometric analyses were conducted on our survey questionnaire, which could impact content validity and contribute to potential interpretation bias of individual survey items. For example, the survey instrument contains items with undefined terms and untested item stems which could have been confusing to respondents or interpreted differently. The findings may have been impacted by response bias with a total 39.4% response rate. The response rate might have been improved with use of an incentive. This survey probed topics which may be considered controversial or highly emotionally driven within the chiropractic field. There is potential for resulting social desirability bias and a need to further explore these themes in greater depth. Lastly, the survey item used to identify subgroups was limited to three options. There is the potential for additional subgroups to exist and further ways in which to subgroup the chiropractic profession in the United States.

Despite limitations, this study had several strengths. To our knowledge this is one of the largest surveys related to clinical practice beliefs and behaviors of licensed chiropractors (*n* = 3538) that has been conducted to date. The survey instrument was modeled from prior studies and completed field testing which provided face validity of the survey [2, 13, 19]. The survey design was representative of licensed chiropractors throughout the United States and included a random sample, stratified by state and District of Columbia. The sample in this study is consistent with prior demographic profiles of the chiropractic profession [28, 29].

## Conclusions

Chiropractic has a long history of intra-professional discord surrounding identity, resulting in the presence of multiple subgroups and related subcultures. This cross-sectional survey (*n* = 3538) of licensed chiropractors in the United States revealed unique relationships between three distinct subgroups within the profession in the United States. The majority (56.8%) of chiropractors in this study self-identified with a spine and neuromusculoskeletal focused subgroup. More than one-fifth of chiropractors in this study belonged to a primary care focused subgroup, while another approximately one-fifth identified with a vertebral subluxation focused subgroup.

## Additional file

**Additional file 1: Appendix 1A**. Number of licensed chiropractors, survey mailings, and response rates for each state and Washington, DC. **Appendix 1B**. Chiropractic program attended by respondents. **Appendix 1C**. Percentages of responses to survey items related to practice ideologies and behaviors of licensed chiropractors in the United States. **Appendix 1D**. Multinomial logistic regression models.

## Data Availability

The dataset used and analyzed during the current study are available from the corresponding author upon reasonable request.

## Abbreviations

IAF: Institute for Alternative Futures
DCs: Doctors of Chiropractic
IRB: Institutional Review Board
