# Supplementary material for: Evaluation of United States chiropractic professional subgroups: a survey of randomly sampled chiropractors
Source: BMC Health Serv Res. 2021 Oct 5;21:1049. doi: 10.1186/s12913-021-07081-0 (PMC8491397; doi:10.1186/s12913-021-07081-0)
Supplement: Supplementary file 1 — Additional file 1: Appendix 1A. Number of licensed chiropractors, survey mailings, and response rates for each state and Washington, DC. Appendix 1B. Chiropractic program attended by respondents. Appendix 1C. Percentages of responses to survey items related to practice ideologies and behaviors of licensed chiropractors in the United States. Appendix 1D. Multinomial logistic regression models. [file 12913_2021_7081_MOESM1_ESM.docx]

Appendix 1A. Number of licensed chiropractors, survey mailings, and response rates for each state and Washington, DC.

| **State of Primary Practice** | **# of Licensed Chiropractors** | **# of Surveys Sent** | **Response Rate** | **Percentage of total survey responses** |
| --- | --- | --- | --- | --- |
| Alabama | 800 | 78 | 44.9% | 1.0% |
| Alaska | 330 | 31 | 48.4% | 0.4% |
| Arizona | 2,440 | 223 | 40.4% | 2.5% |
| Arkansas | 560 | 54 | 38.9% | 0.6% |
| California | 13,170 | 1,252 | 30.0% | 10.5% |
| Colorado | 2,820 | 242 | 33.1% | 2.2% |
| Connecticut | 990 | 104 | 55.8% | 1.6% |
| Delaware | 210 | 28 | 35.7% | 0.3% |
| Florida | 6,150 | 612 | 36.3% | 6.2% |
| Georgia | 3,350 | 307 | 32.2% | 2.8% |
| Hawaii | 550 | 38 | 28.9% | 0.3% |
| Idaho | 740 | 76 | 42.1% | 0.9% |
| Illinois | 3,920 | 407 | 38.6% | 4.4% |
| Indiana | 1,380 | 132 | 49.2% | 1.8% |
| Iowa | 1,880 | 170 | 48.8% | 2.3% |
| Kansas | 1,180 | 129 | 42.6% | 1.5% |
| Kentucky | 890 | 98 | 43.9% | 1.2% |
| Louisiana | 210 | 28 | 50.0% | 0.4% |
| Maine | 360 | 40 | 52.5% | 0.6% |
| Maryland | 690 | 85 | 34.1% | 0.8% |
| Massachusetts | 1,800 | 155 | 46.5% | 2.0% |
| Michigan | 1,750 | 187 | 36.4% | 1.9% |
| Minnesota | 3,090 | 302 | 43.7% | 3.7% |
| Mississippi | 300 | 32 | 46.9% | 0.4% |
| Missouri | 2,410 | 218 | 42.7% | 2.6% |
| Montana | 420 | 39 | 61.5% | 0.7% |
| Nebraska | 730 | 83 | 43.4% | 1.0% |
| Nevada | 680 | 75 | 33.3% | 0.7% |
| New Hampshire | 390 | 44 | 56.8% | 0.7% |
| New Jersey | 3,280 | 317 | 38.8% | 3.4% |
| New Mexico | 500 | 47 | 48.9% | 0.6% |
| New York | 5,160 | 541 | 45.8% | 7.0% |
| North Carolina | 2,070 | 198 | 38.9% | 2.2% |
| North Dakota | 430 | 45 | 51.1% | 0.6% |
| Ohio | 2,490 | 247 | 47.0% | 3.3% |
| Oklahoma | 840 | 81 | 37.0% | 0.8% |
| Oregon | 1,570 | 166 | 57.2% | 2.7% |
| Pennsylvania | 4,130 | 383 | 39.7% | 4.3% |
| Rhode Island | 260 | 36 | 52.8% | 0.5% |
| South Carolina | 1,030 | 97 | 42.3% | 1.1% |
| South Dakota | 430 | 45 | 53.3% | 0.7% |
| Tennessee | 1,100 | 125 | 41.6% | 1.5% |
| Texas | 5,730 | 581 | 29.6% | 4.8% |
| Utah | 970 | 83 | 42.2% | 1.0% |
| Vermont | 260 | 21 | 76.2% | 0.4% |
| Virginia | 1,760 | 149 | 45.6% | 1.9% |
| Washington | 2,430 | 258 | 41.9% | 3.0% |
| West Virginia | 320 | 30 | 40.0% | 0.3% |
| Wisconsin | 2,140 | 226 | 49.1% | 3.1% |
| Wyoming | 180 | 20 | 65.0% | 0.4% |
| Washington, DC | 90 | 7 | 42.9% | <0.1% |

Appendix 1B. Chiropractic program attended by respondents.

| **Chiropractic College Attended** | **Percentage of total survey responses** |
| --- | --- |
| Cleveland College of Chiropractic – Kansas City | 4.3% |
| Cleveland College of Chiropractic – Los Angeles | 1.4% |
| Canadian Memorial Chiropractic College | 0.2% |
| D’Youville College | 0.1% |
| Keiser University | <0.1% |
| Life University | 12.0% |
| Life University West | 2.7% |
| Logan University | 9.4% |
| National University of Health Sciences | 7.4% |
| Northwestern University of Health Sciences | 6.8% |
| New York Chiropractic College | 8.4% |
| Palmer College of Chiropractic – Iowa | 20.8% |
| Palmer College of Chiropractic – Florida | 1.1% |
| Palmer College of Chiropractic – West | 2.8% |
| Parker University | 4.8% |
| Southern California University of Health Sciences | 5.5% |
| Sherman College of Chiropractic | 2.0% |
| Texas Chiropractic College | 2.5% |
| University of Bridgeport | 1.2% |
| University of Western States | 4.7% |
| Multiple | 0.6% |
| Other | 0.6% |

Appendix 1C. Percentages of responses to survey items related to practice ideologies and behaviors of licensed chiropractors in the United States.

| **(Survey item #1) In the examination and assessment of a patient’s condition, do you perform** | **Percentage** |
| --- | --- |
| Only perform spinal analysis to assess for presence of vertebral subluxation | 9.0% |
| Only perform differential diagnosis | 7.7% |
| Usually perform spinal analysis to assess for presence of vertebral subluxation, sometimes perform differential diagnosis | 3.2% |
| Usually perform differential diagnosis, sometimes perform spinal analysis to assess for presence of vertebral subluxation | 1.6% |
| Always perform spinal analysis to assess for presence of vertebral subluxation and differential diagnosis | 78.5% |
| **(Survey item #2)) Conditions you treat** |  |
| Broadest spectrum of health concerns which may include lifestyle and wellness issues | 28.7% |
| Neuromusculoskeletal problems such as low back and neck-related pain | 29.9% |
| General problems and biomechanical groups | 10.9% |
| Biomechanical group and organic/visceral complaints | 5.1% |
| Vertebral subluxation as a somatic joint dysfunction and/or related to functional or musculoskeletal problems | 8.1% |
| Vertebral subluxation as an encumbrance to expression of health | 17.2% |
| **(Survey item #3) Best role for chiropractors in the greater healthcare system** |  |
| Subluxation detection & removal | 21.2% |
| General primary care | 22.0% |
| Spine/neuromusculoskeletal care | 56.8% |
| **(Survey item #4) Chiropractic spinal adjustments could be used to help patients with cancer by** |  |
| Removing interference to innate intelligence | 11.3% |
| Improving nervous system & immune system function | 41.6% |
| Reducing pain & improving movement, quality of life | 43.1% |
| No role for spinal adjustments for people with cancer | 4.1% |
| **(Survey item #5) Vaccinations have had a positive effect on global public health** |  |
| Strongly agree | 11.6% |
| Agree | 28.1% |
| Neutral | 22.3% |
| Disagree | 19.7% |
| Strongly Disagree | 18.3% |
| **(Survey item #6) X-ray is helpful in detection of vertebral subluxations** |  |
| Strongly agree | 22.5% |
| Agree | 31.2% |
| Neutral | 20.8% |
| Disagree | 15.7% |
| Strongly Disagree | 9.8% |
| **(Survey item #7) Percentage of new patients that you take or order x-rays** |  |
| 0-20% | 38.7% |
| 21-40% | 18.8% |
| 41-60% | 10.9% |
| 61-80% | 11.4% |
| 81-100% | 20.3% |

Appendix 1D. Multinomial logistic regression models.

|  | Subluxation focused | Spine/NMSK focused | | | Primary care focused | | |
| --- | --- | --- | --- | --- | --- | --- | --- |
| Q1: Scope of Examination^1^ | BASE OUTCOME | *p* | 95% CI | | *p* | 95% CI | |
| SA |  | -- | -- | -- | -- | -- | -- |
| SA>DDx |  | <.001 | 1.85 | 5.61 | .32 | .62 | 4.42 |
| SA+DDx |  | <.001 | 9.02 | 16.57 | <.001 | 10.98 | 30.57 |
| DDx>SA |  | <.001 | 14.36 | 273.96 | <.001 | 17.12 | 420.80 |
| DDX |  | <.001 | 57.27 | 450.01 | <.001 | 51.01 | 494.97 |
| Gender |  | .52 | .75 | 1.16 | .12 | .95 | 1.53 |
| State |  | .74 | 1.00 | 1.01 | .04 | .99 | 1.00 |
| Chiropractic College |  | .17 | .99 | 1.04 | .45 | .98 | 1.04 |
| Years in Practice |  | .56 | .94 | 1.11 | <.001 | 1.11 | 1.35 |
| Survey Type |  | .91 | .75 | 1.38 | .72 | .75 | 1.53 |
| constant |  | <.001 | .16 | .37 | .00 | .03 | .11 |
| Q2: Conditions treated^2^ | BASE OUTCOME | *p* | 95% CI | | *p* | 95% CI | |
| VS |  | -- | -- | -- | -- | -- | -- |
| Broad |  | <.001 | 15.21 | 29.82 | <.001 | 30.29 | 65.71 |
| Somatovisc |  | <.001 | 12.98 | 39.06 | <.001 | 14.59 | 48.55 |
| MSKsub |  | <.001 | 8.42 | 17.63 | <.001 | 1.62 | 5.07 |
| MSKgen |  | <.001 | 45.12 | 129.69 | <.001 | 19.16 | 63.62 |
| nMSK |  | <.001 | 109.07 | 266.76 | <.001 | 12.32 | 36.66 |
| Gender |  | .64 | .73 | 1.21 | .90 | .76 | 1.27 |
| State |  | .24 | 1.00 | 1.01 | .22 | .99 | 1.00 |
| Chiropractic College |  | .51 | .96 | 1.02 | .51 | .96 | 1.02 |
| Years in Practice |  | .32 | .95 | 1.16 | .01 | 1.03 | 1.28 |
| Survey Type |  | .70 | .78 | 1.46 | .93 | .71 | 1.45 |
| constant |  | <.001 | .10 | .25 | <.001 | .59 | .17 |

**NMSK**: Neuromusculoskeletal

^1^ Survey Question 1 labels: **SA:** Spinal analysis to detect subluxation only; **SA>DDx:** Focus on Spinal analysis, sometimes includes differential diagnosis; **SA+DDx:** Equal focus on spinal analysis to detect subluxation and differential diagnosis; **DDx>SA:** Focus on differential diagnosis, sometimes includes spinal analysis; **DDx:** Differential Diagnosis only

^2^ Survey Question 2 labels: **VS:** Vertebral Subluxation as an Encumbrance to Health; **Broad:** Broad Spectrum of Health Concerns Including Lifestyle and Wellness Issues; **Somatovisc:** Biomechanical and Organic/Visceral Conditions; **MSKsub:** Vertebral Subluxation as a Musculoskeletal Condition; **MSKgen:** General and Biomechanical Conditions; **nMSK:** Neuromusculoskeletal Conditions

|  | Subluxation focused | Spine/NMSK focused | | | Primary care focused | | |
| --- | --- | --- | --- | --- | --- | --- | --- |
| Q4: Role of SMT in Cancer Treatment^3^ | BASE OUTCOME | *p* | 95% CI | | *p* | 95% CI | |
| Innate |  | -- | -- | -- | -- | -- | -- |
| ImmuneFx |  | .00 | 5.09 | 9.52 | .00 | 3.63 | 7.14 |
| QoL |  | .00 | 38.19 | 79.75 | .00 | 10.26 | 22.85 |
| None |  | .00 | 18.24 | 71.91 | .00 | 2.60 | 13.89 |
| Gender |  | .42 | .73 | 1.14 | .29 | .90 | 1.43 |
| State |  | .17 | 1.00 | 1.01 | .18 | .99 | 1.00 |
| Chiropractic College |  | .66 | .98 | 1.03 | .62 | .98 | 1.03 |
| Years in Practice |  | .27 | .96 | 1.15 | .00 | 1.13 | 1.37 |
| Survey Type |  | .11 | .62 | 1.05 | .05 | .55 | 1.01 |
| constant |  | .00 | .13 | .32 | .00 | .10 | .26 |
| Q5: Vaccination | BASE OUTCOME | *p* | 95% CI | | *p* | 95% CI | |
| Strongly agree |  | -- | -- | -- | -- | -- | -- |
| Agree |  | .00 | .13 | .52 | .14 | .26 | 1.21 |
| Neutral |  | .00 | .04 | .17 | .00 | .10 | .44 |
| Disagree |  | .00 | .02 | .09 | .00 | .08 | .34 |
| Strongly disagree |  | .00 | .01 | .03 | .00 | .05 | .24 |
| Gender |  | .48 | .87 | 1.34 | .03 | 1.02 | 1.62 |
| State |  | .36 | 1.00 | 1.01 | .06 | .99 | 1.00 |
| Chiropractic College |  | .22 | .99 | 1.04 | .26 | .99 | 1.04 |
| Years in Practice |  | .79 | .91 | 1.07 | .00 | 1.07 | 1.29 |
| Survey Type |  | .45 | .71 | 1.17 | .155 | .60 | 1.08 |
| constant |  | .00 | 13.7 | 59.87 | .00 | 1.66 | 8.28 |

**NMSK**: Neuromusculoskeletal
 ^3^ Survey Question 4 labels: **Innate:** Removing Interference to Innate Intelligence; **ImmuneFx:** Improving Nervous System/Immune System Function; **QoL:** Improving Pain/Quality of Life; **None:** No Role

|  | Subluxation focused | Spine/NMSK focused | | | Primary care focused | | |
| --- | --- | --- | --- | --- | --- | --- | --- |
| Q6: Subluxation Detection | BASE OUTCOME | *p* | 95% CI | | *p* | 95% CI | |
| Strongly agree |  | -- | -- | -- | -- | -- | -- |
| Agree |  | .00 | 2.13 | 3.36 | .00 | 1.80 | 3.17 |
| Neutral |  | .00 | 3.10 | 5.40 | .00 | 3.34 | 6.39 |
| Disagree |  | .00 | 5.65 | 11.34 | .00 | 3.85 | 8.69 |
| Strongly Disagree |  | .00 | 7.76 | 20.26 | .00 | 3.73 | 11.39 |
| Gender |  | .03 | .65 | .98 | .50 | .87 | 1.33 |
| State |  | .90 | 1.00 | 1.00 | .03 | .99 | 1.00 |
| Chiropractic College |  | .39 | .99 | 1.03 | .57 | .98 | 1.03 |
| Years in Practice |  | .29 | .96 | 1.13 | .00 | 1.12 | 1.34 |
| Survey Type |  | .01 | .56 | .91 | .01 | .51 | .93 |
| constant |  | .64 | .66 | 1.30 | .00 | .23 | .52 |
| Q7: % of New Patient X-Rays | BASE OUTCOME | *p* | 95% CI | | *p* | 95% CI | |
| 0-20% |  | -- | -- | -- | -- | -- | -- |
| 21-40% |  | .00 | 1.23 | 2.25 | .15 | .91 | 1.84 |
| 41-60% |  | .32 | .85 | 1.65 | .32 | .55 | 1.22 |
| 61-80% |  | .26 | .62 | 1.14 | .01 | .40 | .85 |
| 81-100% |  | .00 | .27 | .42 | .00 | .24 | .43 |
| Gender |  | .02 | .65 | .95 | .69 | .85 | 1.29 |
| State |  | .96 | 1.00 | 1.00 | .01 | .99 | 1.00 |
| Chiropractic College |  | .06 | 1.00 | 1.04 | .26 | .99 | 1.04 |
| Years in Practice |  | .61 | .91 | 1.06 | .00 | 1.08 | 1.29 |
| Survey Type |  | .00 | .44 | .73 | .00 | .42 | .79 |
| constant |  | .00 | 2.31 | 4.55 | .30 | .83 | 1.85 |

**NMSK**: Neuromusculoskeletal
